# Cutting-based single atomic layer removal mechanism of monocrystalline copper: edge radius effect

**DOI:** 10.1186/s11671-019-3195-4

**Published:** 2019-12-06

**Authors:** Wenkun Xie, Fengzhou Fang

**Affiliations:** 1 Centre of Micro/Nano Manufacturing Technology (MNMT-Dublin), University College Dublin, Dublin, Ireland; 2 State Key Laboratory of Precision Measuring Technology and Instruments, Centre of Micro/Nano Manufacturing Technology (MNMT), Tianjin University, Tianjin, China

**Keywords:** Single atomic layer removal, Cutting edge radius effect, Atomic sizing effect, Mechanical cutting, Dislocation motion

## Abstract

The ultimate objective of mechanical cutting is to down minimum chip thickness to single atomic layer. In this study, the cutting-based single atomic layer removal mechanism on monocrystalline copper is investigated by a series of molecular dynamics analysis. The research findings report that when cutting depth decreases to atomic scale, minimum chip thickness could be down to single atomic layer by mechanical cutting using rounded edge tool. The material removal behaviour during cutting-based single atomic layer removal exhibits four characteristics, including chip formation by shearing-stress driven dislocation motion, elastic deformation on the processed surface, atomic sizing effect, and cutting-edge radius effect. Based on this understanding, a new cutting model is proposed to study the material removal behaviour in cutting-based single atomic layer removal process, significantly different from those for nanocutting and conventional cutting. The outcomes provide theoretical support for the research and development of the atomic and close-to-atomic scale manufacturing technology.

## Introduction

Mechanical cutting, as one of most important subtractive machining methods, has been applied to produce parts with a high surface finish quality [1, 2]. A large number of theoretical and experimental studies have been carried out to clarify the underling material removal mechanism at nanoscale to establish and enrich the basic nanocutting theory [3–6]. The research outcomes significantly contribute to the application of nanometric cutting in academia and industry, enabling the manufacturing of high-performance parts requiring complex form and nanometric surface finish quality [7, 8]. However, there is still no report on the mechanism of material removal in cutting at atomic and close-to-atomic scale (ACS), seriously restricting the progress of developing next-generation manufacturing technology—atomic and close-to-atomic scale manufacturing (ACSM), i.e., Manufacturing III [9]. Moreover, the progressive development of atomic scale devices also stressed the demand for various machining processes to achieve ACSM [10].

In conventional macroscale cutting, cutting depth is significantly larger than cutting edge radius, where the material removal is realized by material shearing-driven chip formation [11–13]. In micro/nanocutting, as cutting depth is comparable or lower than tool edge radius, the chip formation of extrusion gradually becomes dominate with cutting depth decreasing down to nanoscale, which is greatly influenced by the cutting-edge radius effects [14–19]. In ACS cutting, as the cutting depth is further decreased to close-to-atomic scale and even atomic scale, which is much lower than cutting edge radius, the edge radius effect would inevitably affect the material removal behaviour.

Moreover, unlike in conventional cutting and micro/nano cutting, in ACS cutting, cutting depth is not only significantly lower than cutting edge radius, but also comparable or even lower than the radius of the workpiece atoms. The practical material removal behaviour would be changed at different ratios of cutting depth to workpiece atomic radius, which is recognized as one new sizing effect, i.e., atomic sizing effect [20]. Therefore, in ACS cutting, both cutting-edge radius effect and atom sizing effect should be considered. However, there is no report about this critically important issue in the study of ACS cutting technology.

For those reasons, in the present study, the atomic sizing effect and edge radius effect on cutting-based single atomic layer removal mechanism are investigated by using molecular dynamics(MD) modelling.

This paper is structured by following sections. Section 2 introduces the methodology used, including modelling and protocol, suitable potential function. Section 3 presents the analysis results and relevant discussions. Section 4 discusses the detailed cutting-based single atomic layer removal mechanism, under the coupled influence of cutting-edge effect and atomic sizing effect. The study findings are concluded in the Section 5.

## Methodology

### Simulation Model and Protocol

As cutting depth decreases to atomic or close-to-atomic scale, it is indeed challenging to experientially observe the material removal process in practice. In this study, a series of MD simulations are conducted to analyse cutting-based single atomic layer removal mechanism, emphatically focusing the workpiece atomic sizing effect and cutting-edge radius effect.

**Fig. 1:**
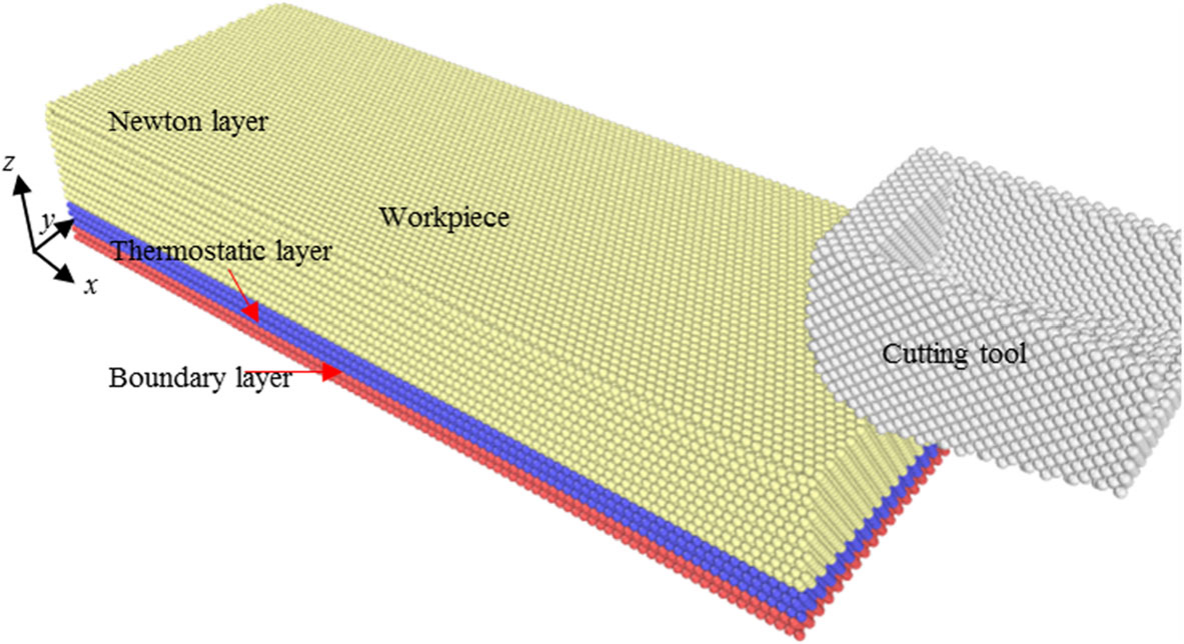
Simulation model for diamond-cutting model

Figure 1 shows the snapshots for atomic configuration of diamond-copper cutting model, which consists of a single-crystal copper workpiece and a diamond cutting tool. The simulations are conducted on the (111) plane of single crystal copper. The dimensions of the workpiece in *x*-[1 -1 0], *y*-[1 1 -2], and *z*-[1 1 1] directions are 27, 10, and 5 nm, respectively. During MD simulations, the workpiece atoms are divided into boundary layer atoms, thermostatic layer atoms, and Newtonian layer atoms, respectively, as shown in Fig. 1. The two layers at the bottom of the workpiece, namely, boundary layer, are kept fixed over the MD simulations, to eliminate the possible cutting-induced position translation of workpiece. The three atomic layers adjacent to boundary layer are thermostat layer atoms. The temperature of thermostatic layer is kept at 298 K by velocity rescaling methods. The remaining workpiece atoms belong to Newtonian layer. In the present simulations, the rake angle and clearance angle of the cutting tool is 0° and 12°. The detailed model parameters are summarized in Table 1. Since ACS cutting is preferably conducted at a lower cutting velocity, a cutting velocity of 25 m/s along [-1 1 0] direction is used after considering the effect of cutting velocity and computational time cost.

**Table 1:** Parameters used in the MD simulation

Configuration	Diamond cutting process
Workpiece material	Single crystal copper
Workpiece	Face-centred-cubic (FCC) structure
Crystal structure	Lattice constant = 3.65 Å
Workpiece size	27 × 10 × 5nm^3^
Tool material	Diamond
Cutting velocity	25 m/s
Tool edge radius	0 nm, 2 nm, 3 nm, 4 nm, 5 nm, 7.5 nm, 10 nm
Tool rake angle	0°
Tool clearance angle	12°

### Potential Function

The copper-diamond cutting system mainly involves two types of atoms, namely, copper, and diamond atoms. In the MD simulations, the interatomic interactions should be accurately described to ensure the computational accuracy of simulation results. Thus, reasonable selection of potential function is critically significant. In this study, the frequently-used embedded atom method (EAM) potential function is adopted to describe the interatomic interactions among copper atoms [21]. Morse function is applied to calculate the interactions between copper atoms and diamond atoms (Cu-C), mainly depending on the *r.*

1 $$ E={D}_0\left[{e}^{-2\alpha \left(r-{r}_0\right)}-2{e}^{\alpha \left(r-{r}_0\right)}\right] $$

where *E* and *D*_0_ refer to pair potential energy and cohesion energy, *α* represents a constant, *r*_*0*_ is the equilibrium distance, and *r* is the distance between two atoms. For Cu-C interactions, *D*_*0*_ is 0.087ev, *r*_*0*_ is 0.205 nm [22], and *α* is 51.40 nm^−1^*.* For the interactions between carbons atoms in cutting tool (C-C), the significantly stronger bond strength between diamond atoms than copper atoms are negligible. The cutting tool is regarded as rigid during the analysis.

### Definition of Cutting Depth

**Fig. 2:**
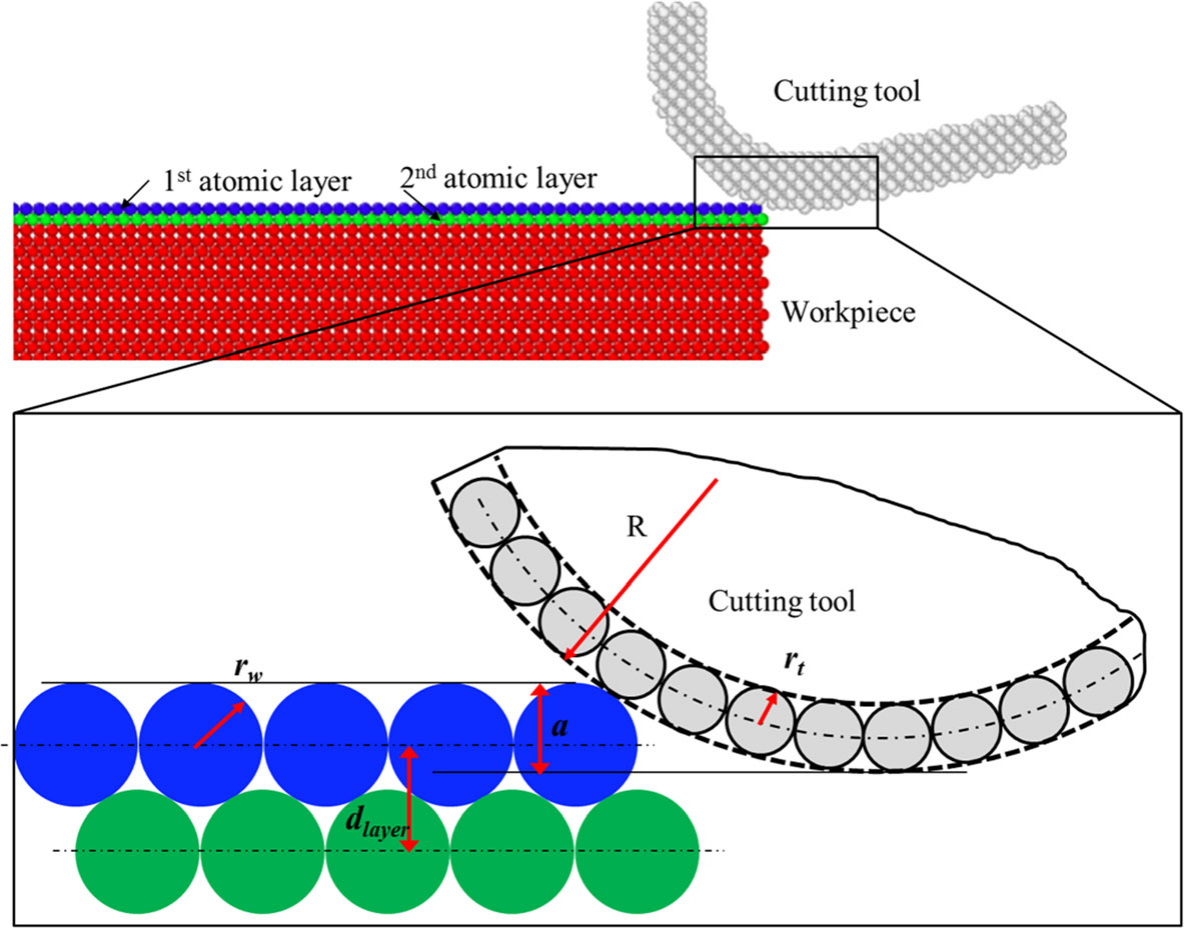
Schematic diagram for the cutting depth definition in single atomic layer removal

Cutting depth (*a*) is defined to be the distance between the topmost point of workpiece surface and the lowest point of cutting tool. The size of a workpiece atom is usually represented by atomic radius (*r*_*w*_*).* As given in Fig. 2, when the topmost first atomic layer is targeted to be removed from the workpiece surface, theoretically, the maximum cutting depth used can be obtained as the following:

2 $$ {a}_{\mathrm{max}}={r}_w+0.5\ast {d}_{layer} $$

Here, *d*_layer_ represents the spacing distance between the topmost neighbouring atomic layers on the workpiece surface. In this study, all analysis is based on the (111) surface of monocrystalline copper workpiece; therefore, the *r*_*w*_ in Eq. (2) refers to the radius of copper atoms, namely, 1.28 Å. The *d*_layer_ is 2.087 Å. When *a* is larger than *a*_max_, the lowest point of cutting tool will come into direct contact with the second atomic layer and may induce the material deformation and even removal. Therefore, in the present study, the cutting depths used are smaller than *a*_max_ (2.32 Å).

Based on the findings, the fundamental cutting-based single atomic layer removal mechanism will be greatly changed at various combinations of the ratios of *a* to *R* and *a* to *r*_*w*_*.*

## Results and Discussion

According to MD results, both atomic sizing effect and cutting-edge radius effect have greatly influenced the cutting-based single atomic layer removal process. In the following sections, to clearly describe the atomic sizing effect and tool edge radius effect, the ratio of cutting depth (*a*) to workpiece atomic radius (*r*_*w*_), *a/r*_*w*_, and that of cutting depth(*a*) to edge radius(*R*), *a/R*, are employed. The analysis results are systematically studied from the aspects of chip formation, surface generation, subsurface deformation and atomic displacement behaviour. The findings provide detailed insights into the typical characteristics in cutting-based single atomic layer removal mechanism.

### Chip Formation

The analysis results indicate that due to the workpiece atomic sizing effect [20], there are two critical values of the ratio of cutting depth (*a*) to workpiece atomic radius (*r*_*w*_), namely, critical value 1 (C_1_) and critical value 2 (C_2_), which divided the chip formation behaviour into different cases.

- The ratio of *a/r*_*w*_ smaller than critical value 1 (C_1_).

**Fig. 3:**
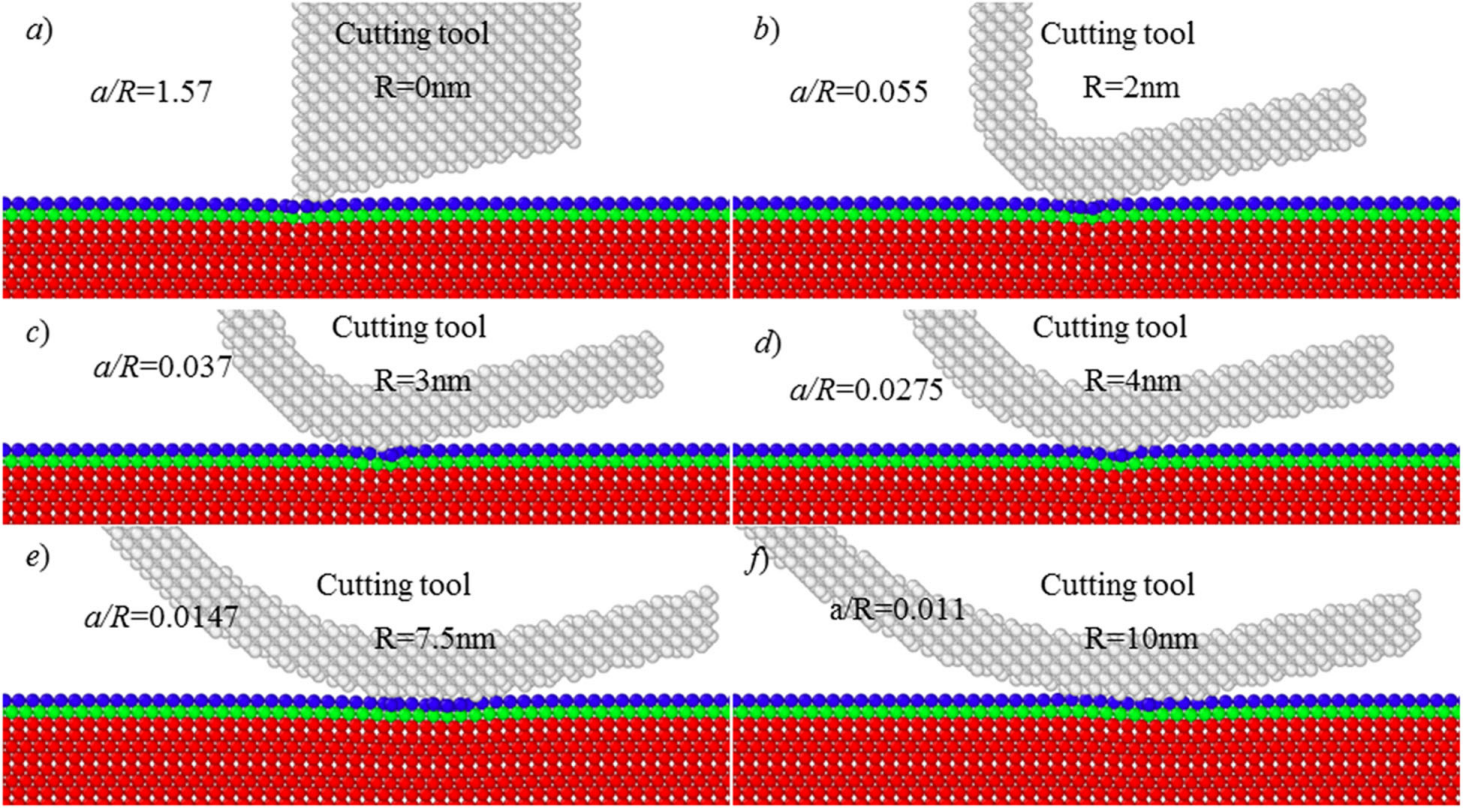
Simulation results at cutting depth of 1.1 Å

Figure 3 shows the results of MD simulation at various tool edge radius at the cutting depth of about 1.1 Å. Here, the ratio of cutting depth (*a*) to workpiece atomic radius (*r*_*w*_) is 0.781.

Despite of the increased cutting-edge radius, there is elastic deformation occurring on the processed each Cu (111) surface. There is no chip formation and material removal over the cutting process. The study shows that the cutting depth should be larger than about 1.1 Å to enable material removal on Cu (111) surface.

b. The ratio of *a/r*_*w*_ is larger than C1, but smaller than critical value 2 (C_2_).

**Fig. 4:**
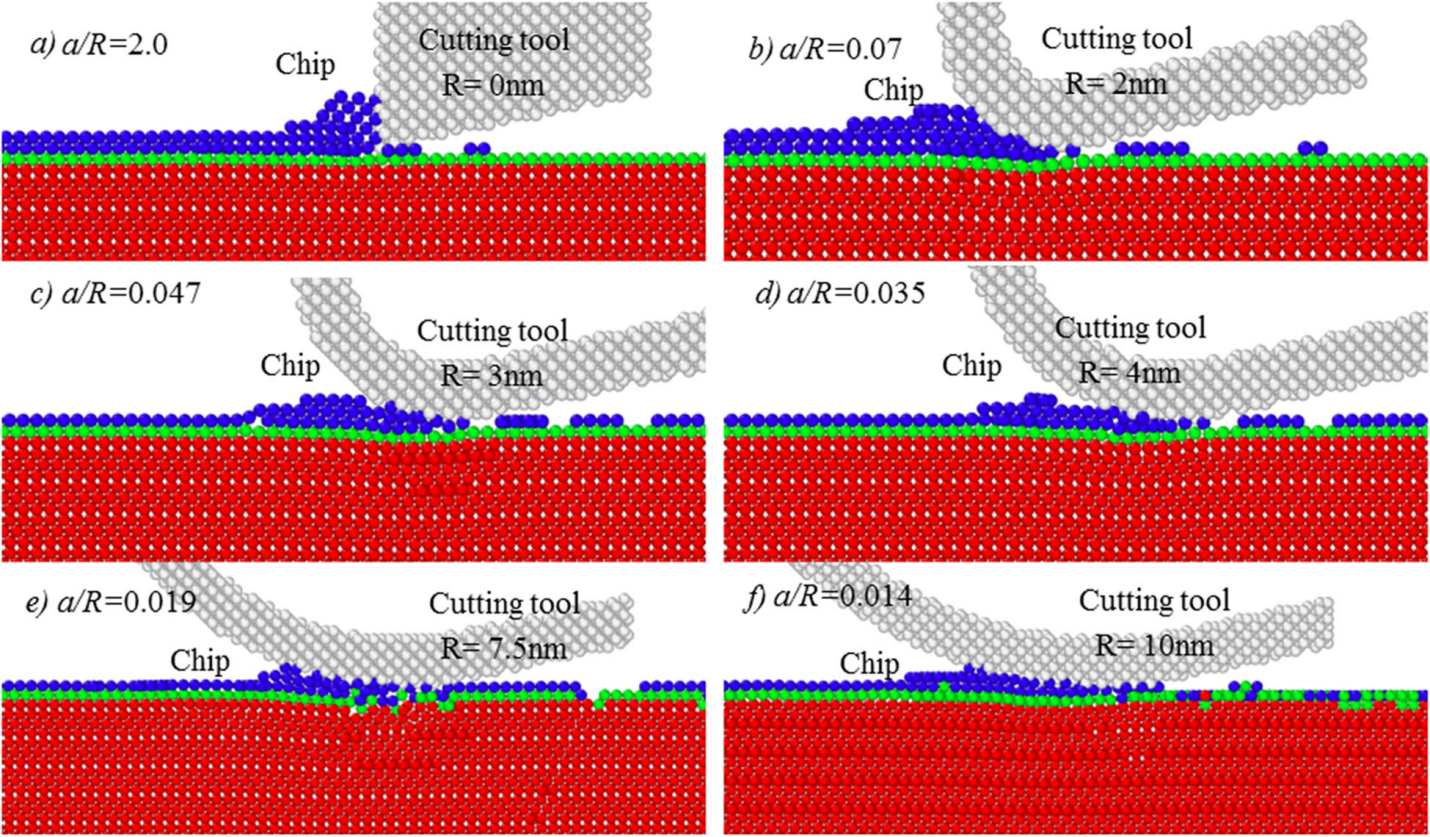
Simulation results at cutting depth of 1.4 Å

When cutting depth is larger than about 1.1 Å, namely, the ratio of *a/r*_*w*_ is larger than 0.781, there is material removal occurring on workpiece surface. As shown in Fig. 4, a part of material is removed from workpiece surface, but a larger number of atoms within the topmost first layer is remained onto the new processed surface, forming surface defects. Moreover, as the edge radius increases to 7.5 nm, the ratio of *a/R* is 0.019. At such case, a part of atoms in first atomic layer has been pressed into second layer and even third atomic layer, as shown in Fig. 4e, which should be ascribed to the extrusion action of cutting tool. It also indicates that tool edge radius effect starts to impose an influence on the material removal process, though a continuous material removal could not be obtained.

c. The ratio of *a/r*_*w*_ is larger than C_2_.

**Fig. 5:**
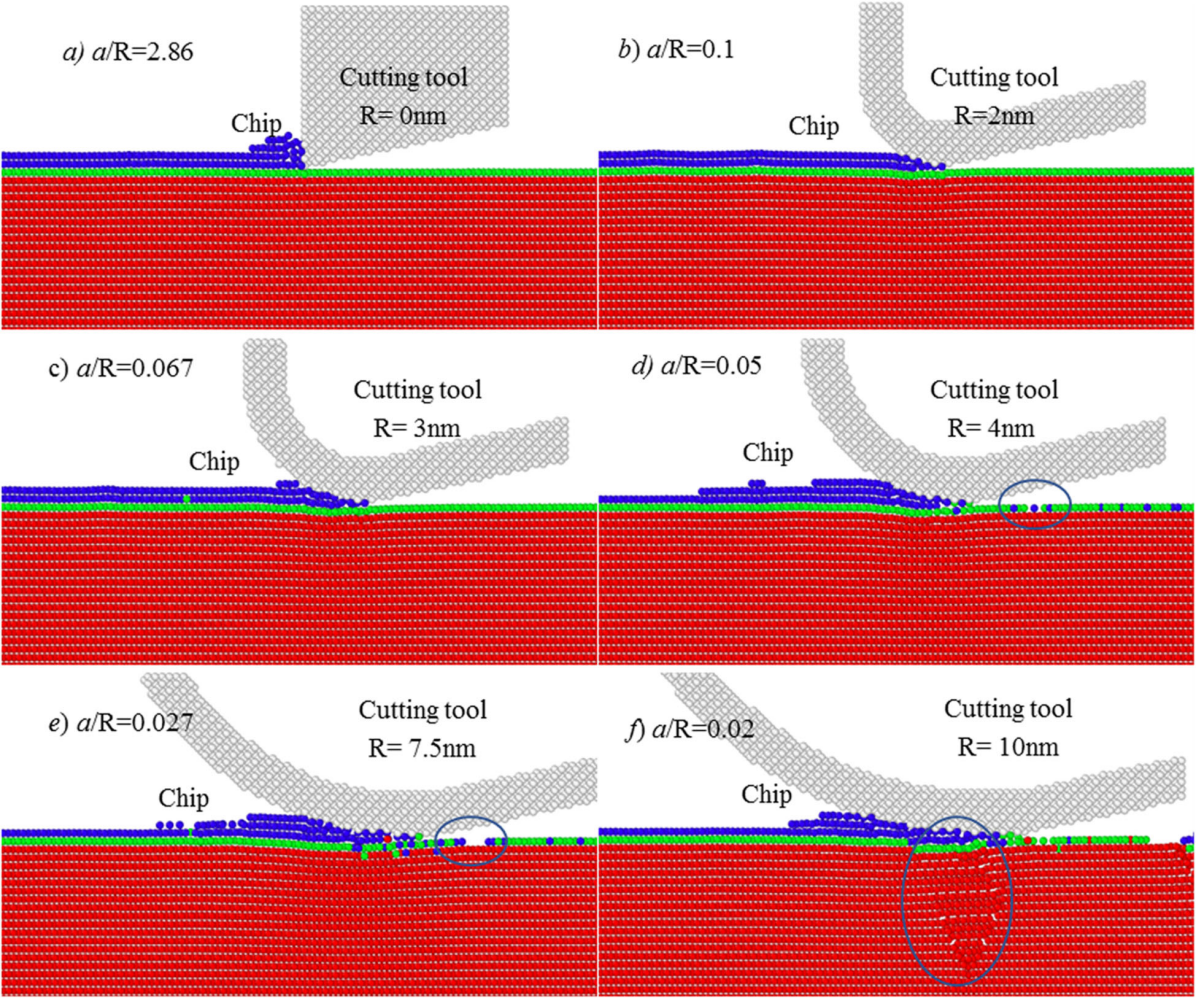
Simulation results of ACS cutting at cutting depth = 2 Å

Figure 5 shows the chip formation at cutting depth of about 2 Å. Here, the ratio of *a/r*_*w*_ = 2 Å/1.28 Å = 1.563. Compared with Fig. 3, with an increase of cutting depth to 2 Å, the atoms within the targeted atomic layer could be continuously and stably removed by chip formation. It indicates that the cutting depth has been larger than the minimum chip thickness of monocrystalline copper, and the minimum chip thickness could be down to single atomic layer with a cutting depth of about 2 Å. After cutting, the materials within first atomic layer were fully removed from workpiece surface.

However, it can be also noticed in Fig. 5 that, due to the increase of the ratio of *a/R*, the atoms in the first atomic layer undergo evidently different removal processes, especially the cutting-induced deformation on the processed surface. At the cutting depth of 2 Å, when sharp cutting tool is used, namely, the ratio of *a/R* is 5.70, only slight deformation occurs on the processed surface. As cutting-edge radius increases to 3 nm, the ratio of *a/R* is 0.134, the amplitude of elastic deformation was evidently increased.

When tool edge radius is increased to 4 nm, as given in Fig. 5d, many atoms within the targeted first atomic layer have been pressed into second atomic layer, forming the new processed surface. At the tool edge radius of about 7.5 nm, a part of atoms in first layer is even diffused to third atomic layer. When tool edge radius reaches about 10 nm, an evident elastic and plastic deformation occurs, which can be also determined in the following Section 3.3.

Therefore, the cutting-based single atomic layer removal depends on not only the ratio of *a/r*_*w*_, but also the ratio of *a/R*. To achieve single atomic layer removal by mechanical cutting, i.e., material removal at atomic scale, both atom sizing effects and cutting-edge radius effect should be considered, significantly different from micro/nanocutting and conventional macroscale cutting.

### Surface Generation

One objective of cutting-based atomic layer removal is to obtain the defect-free processed surface with ideal crystalline structure. The workpiece atomic sizing effect on surface generation in ACS cutting has been studied recently [20]. In the present study, to clearly indicate the cutting-edge radius effect on surface generation in ACS cutting, the surface topography and surface composition of the new processed surface are studied as follows.

#### Surface Topography

**Fig. 6:**
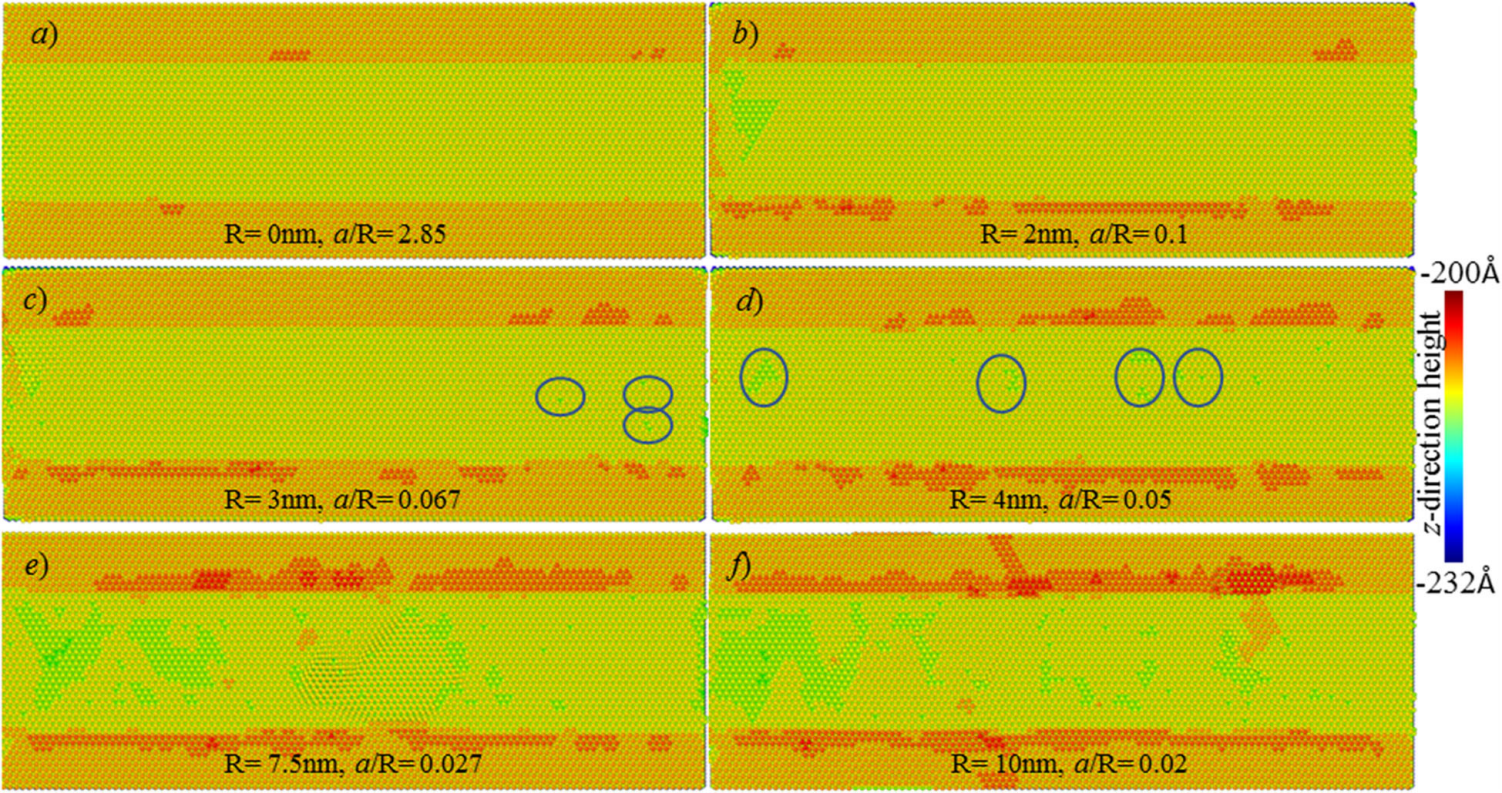
Effects of tool edge radius on surface topographies. Atoms are coloured based on their z-direction heights

Figure 6 shows the surface topographies of the processed Cu (111) surface at different edge radii. Here, a cutting depth of 2 Å is adopted. As shown in Fig. 6a, b, when tool edge radius is smaller than 3 nm, the Cu (111) surfaces with ideal crystalline structure could be obtained. As for the defects at the left-side of workpiece surface, it is due to the deformation during cutting-exit of tool. During the cutting in a stead stage, there is no surface defect formed on the processed surface.

As tool edge radius increases, however, many defects are gradually formed on the processed surface. At the edge radius of 4 nm, many pitted-like defects are formed on the processed surface, see Fig. 6d. Further, when the tool edge radius is equal to or larger than 5 nm, there are a large number of surface defects formed, seriously deteriorating the surface quality. At such case, more than one layer of atoms has been removed from workpiece surface. Therefore, it can be determined that at the cutting depth of 2 Å, when tool edge radius is smaller than 3 nm, single atomic layer removal could be achieved on Cu (111) surface. Due to the cutting-edge radius effect, the ratio of cutting depth (*a*) to edge radius (*R*) should be larger than one threshold, in order to achieve defect-free processed surface via cutting-based single atomic layer removal. Here, the critical value of the ratio of *a/R* is [0.05, 0.067].

#### Surface Composition

**Fig. 7:**
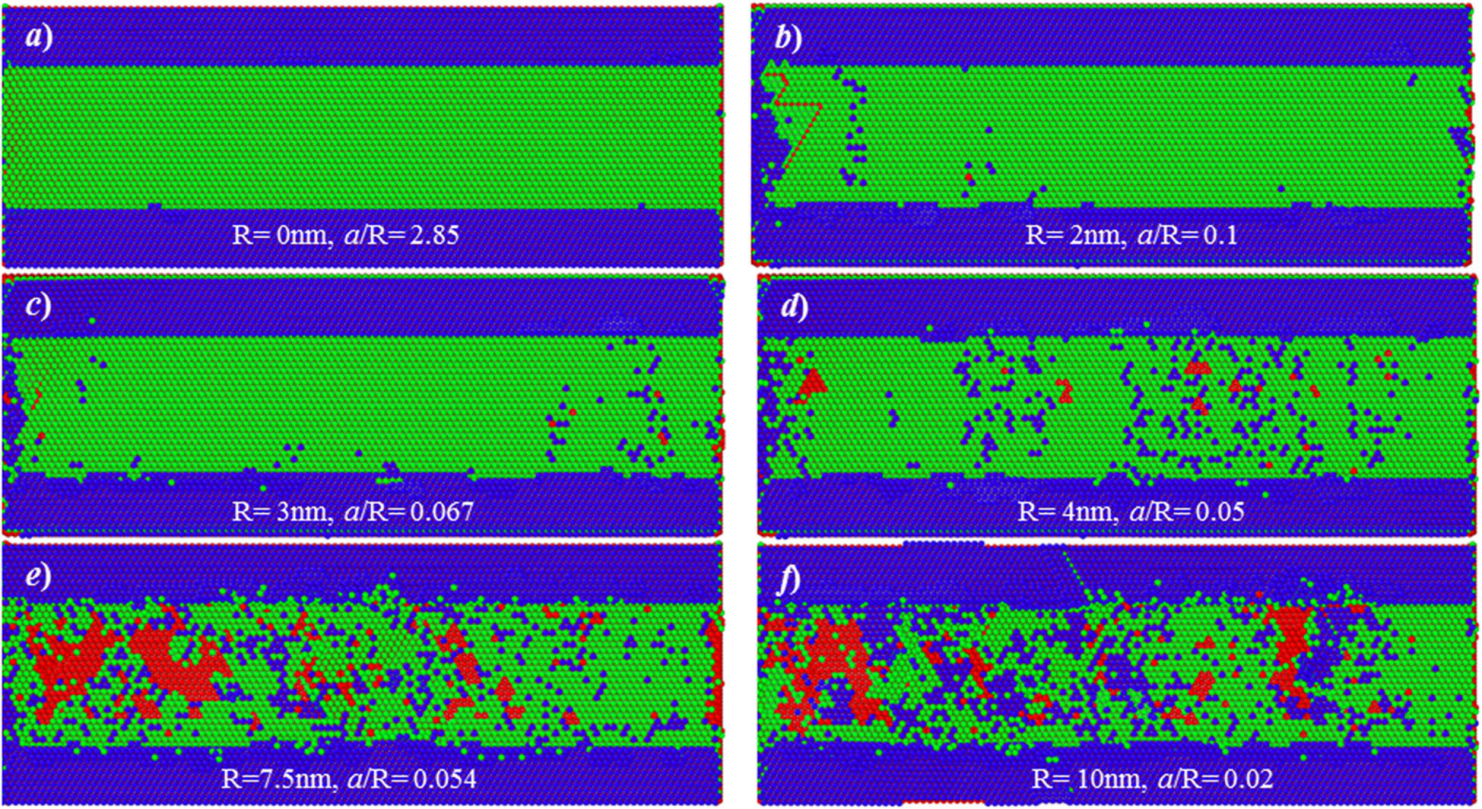
Surface composition of processed surfaces. Atoms are coloured based on the number of atomic layer

Figure 7 shows the composition of the processed surfaces at various tool edge radii and the cutting depth of 2 Å. Here, green and blue atoms are those from first and second layers, while the red atoms are those below second atomic layer. As shown in Fig. 7a, when a sharp cutting tool is used, the ratio of *a/R* is 2.85, the processed surface only consists of atoms in first layer. This result indicates that the targeted first atomic layer has been thoroughly removed from workpiece. Moreover, it means that the material removal is conducted in the form of layer-by-layer, in which the removed materials only comes from the targeted first atomic layer on the workpiece surface.

However, when tool edge radius is 2 nm and 3 nm, as shown in Fig. 7b, c, the processed surfaces are composited of two atomic layers (blue and green). It indicates that though single atomic layer removal has been achieved, the surface generation process involves minimum 2 atomic layers. Further, when tool edge radius is larger than 4 nm, there is a large number of atoms of first atomic layer on the processed surface, indicating that many atoms in first layer have been pressed to generate a new surface.

Therefore, with an increase in cutting edge radius, the surface generation has been greatly changed. Two kinds of surface generation mechanism involved are summarized below:

1. *Layer-by-layer*: the targeted first atomic layer is fully removed to generate new processed surface. Only atoms within first layer are removed during the cutting process.
2. *Multi-layer removal*: though single atomic layer removal could be realized, the atoms within targeted atomic layer undergo two typical displacement behaviours. A part of atoms would be formed into chip by shear stress-driven dislocation motion, while others would be extruded into the processed surface, under the action of cutting tool. The material removal process involves minimum two atomic layers.

### Subsurface Deformation Mechanism

**Fig. 8:**
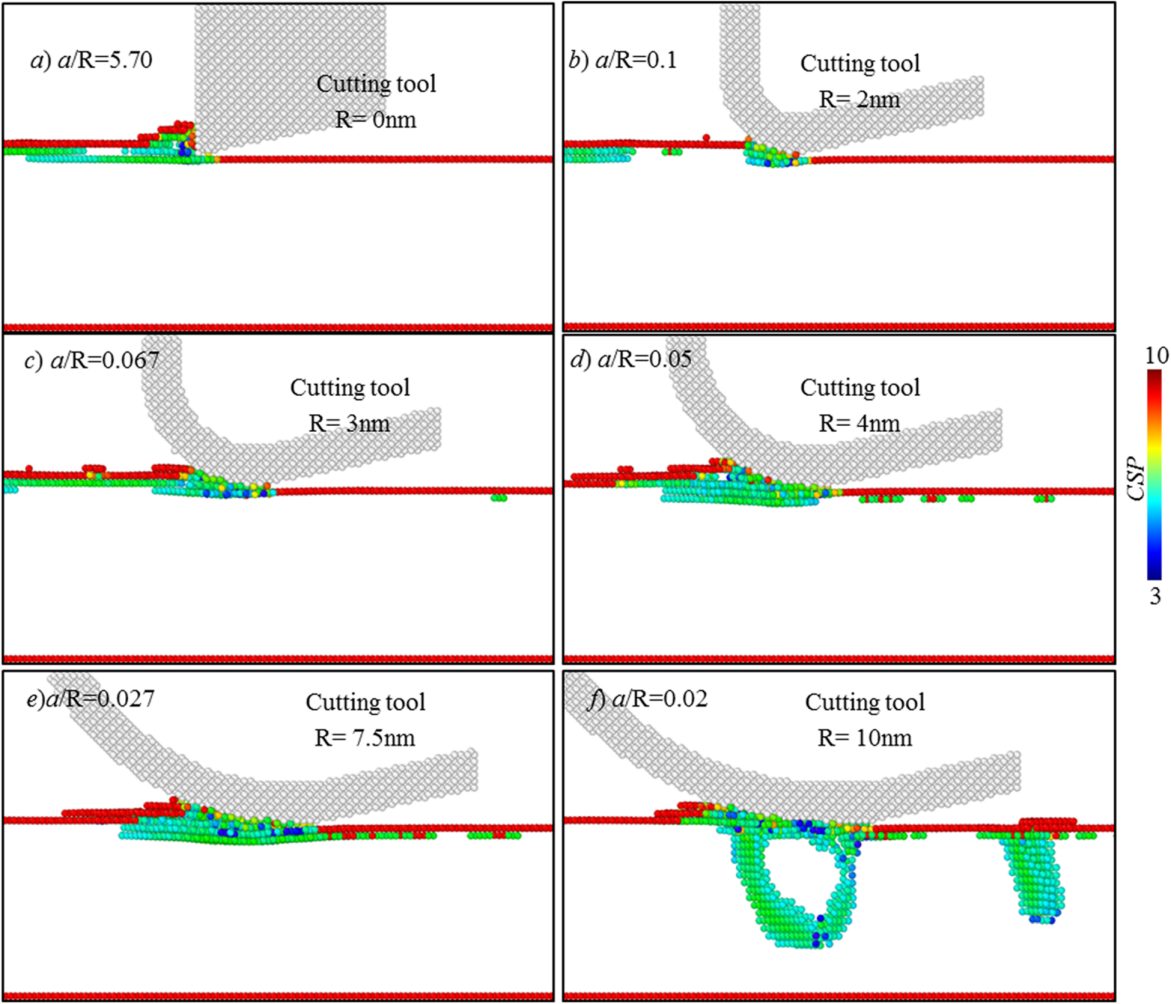
Surface composition of the processed surfaces. Atoms are coloured based on their CSPs

In nanocutting, there is elastic and plastic deformation on the processed surface during cutting process. After the cutting tool passes over workpiece surface, the elastic portion springs back, while the plastic deformed part would lead to lasting deformation [1, 2]. As cutting depth decreases to atomic scale, in the cutting towards single atomic layer removal, it is postulated that there is only elastic deformation occurring on the processed surface. To verify it, the workpiece subsurface deformation states during and after cutting are analysed. Figure 8 illustrates the defect structures in workpiece subsurface at various tool edge radii. Here, the atoms are coloured based on centro-symmetry parameter (CSP), and the atoms with the CSP of smaller than 3 are omitted, which represent those with perfect FCC structure.

When cutting edge radius is smaller than 4 nm, no subsurface defect is formed in the processed surface. There is only elastic deformation on the processed surface in cutting.

**Fig. 9:**
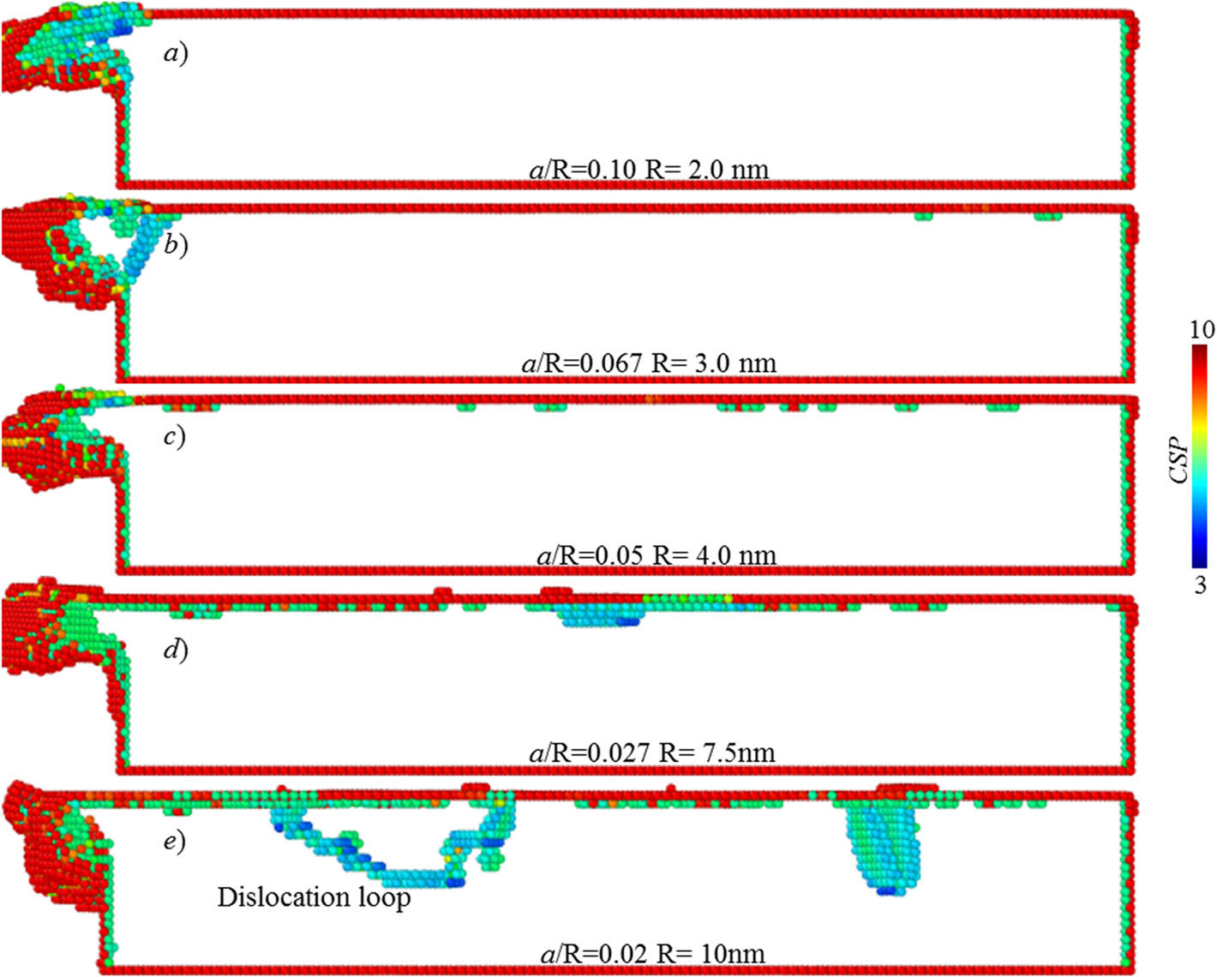
Subsurface defects at various tool edge radii. Atoms are coloured based on their CSPs

As shown in Fig. 8, when cutting edge radius is equal to or larger than 4 nm, there are subsurface defects initialized. Moreover, as cutting-edge radius increase, the number of subsurface defects is significantly increased. When the edge radius reaches about 10 nm, one dislocation loop has been formed, and it could not disappear after cutting, as shown in Fig. 9e. It clearly indicates the plastic deformation occurring on the processed surface.

After tool passes over the workpiece surface, the elastically deformed portion would spring back; thus, parts of subsurface defects are annihilated. As displayed in Fig. 9a–c, finally, there is no subsurface defect existed, when the tool edge radius is 2 nm or 3 nm.

As for the plastically deformed portion, it leads to a lasting deformation. As shown in Fig. 9, when tool edge radius is 7.5 nm, there is small number of subsurface defects remained. When tool edge radius is 10 nm, the dislocation loop and stacking fault are finally existed in the workpiece subsurface.

Based on above analysis, it can be inferred that to enable cutting-based single atomic layer removal, plastic deformation should be avoided, and only elastic deformation is allowed on the processed surface. It is regarded as one characteristic feature of the cutting-based single atomic layer removal process.

### Atomic Displacement Behaviour

**Fig. 10:**
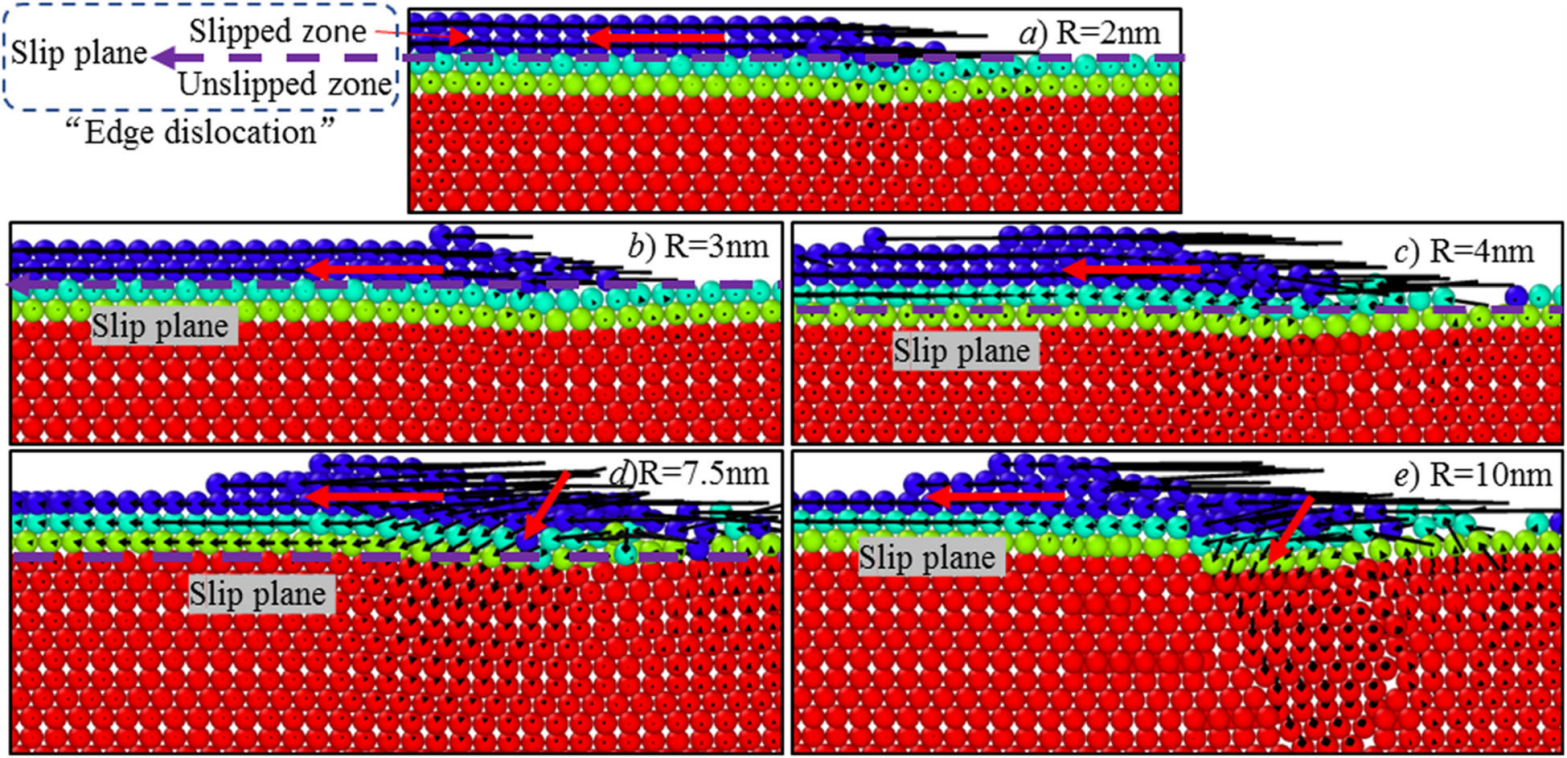
Atomic displacement behaviour at various cutting-edge radius

According to MD trajectory files, dislocation motion has dominated the cutting-based single atomic layer removal process. Figure 10 shows the simulation results using different tool edge radiuses. At the edge radius of 2 nm, under the action of cutting tool, only first atomic layer is slipped along cutting direction to form into chip, while others remain immobilized. It could be regarded as one cross section of one edge dislocation.

As the tool edge radius increases, part of material within first atomic layer has been pressed into the processed surface, inducing the slip of the atomic layers below the first layer. Moreover, as tool edge radius increases, the number of atomic layers undergoing material slip process tends to grow. When tool edge radius is 4 nm, except for first atomic layer, the second atomic layer also conducts material slip along cutting direction, as illustrated in Fig. 10. When the tool edge radius is 7.5 nm, as cutting tool advances, the topmost three atomic layers on the workpiece surface have slipped along cutting direction. Further, when tool edge radius is increased to 10 nm, a large number of materials has been pressed to form new processed surface; there is plastic deformation (see Fig. 10e) occurring on the workpiece surface, which can be also determined in Fig. 9.

**Fig. 11:**
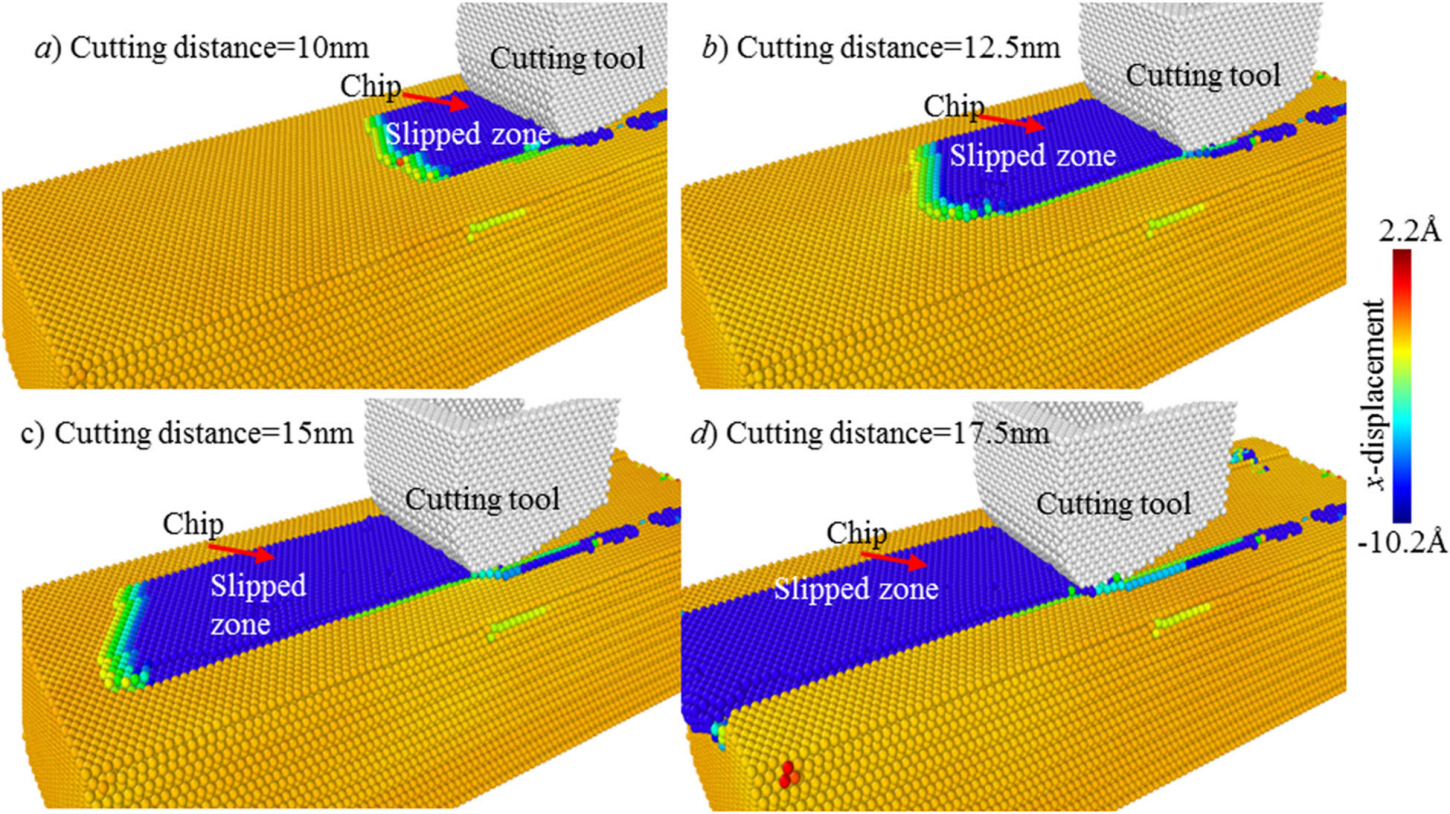
Slip process of the targeted atomic layer at cutting edge radius of 2 nm. Atoms are coloured based on their x-direction atomic displacement values

Figure 11 shows the slip process of the targeted atomic layer along cutting direction. The area of the slipped zone is continually enlarged with the cutting tool moving forward. At the cutting distance of 17.5 nm, the slipped zone has reached a maximum value. Subsequently, the materials within the slipped zone are continuously formed into chip; the volume of chip is also increased.

Overall, as per the analysis in Sections 3.3 and 3.4, as tool edge radius increases, both subsurface deformation mechanism and atomic displacement behaviour has greatly changed. At different tool edge radii, different numbers of atomic layers tend to slip along cutting direction, while different deformation regimes occur on the processed surface, as summarized in Table 2. Moreover, it can be found that in the cutting-based single atomic layer removal process, the chip formation is governed by shear-stress driven dislocation motion, significantly different from the chip formation in conventional cutting and nanocutting, It can be also regarded as one characteristic feature of cutting-based single atomic layer removal.

**Table 2:** Atom displacement behaviour versus cutting edge radius

	Number of slipped atomic layers	Deformation regimes on the processed surface
*R* < 3 nm	1	Elastic deformation
*R* > 3 nm and *R* < 7.5 nm	2	
*R* > 7.5 nm and *R* < 10 nm	3	
*R* > =10 nm	4	Elastic-plastic deformation

### Cutting Force

#### Influence of Edge Radius Effect

**Fig. 12:**
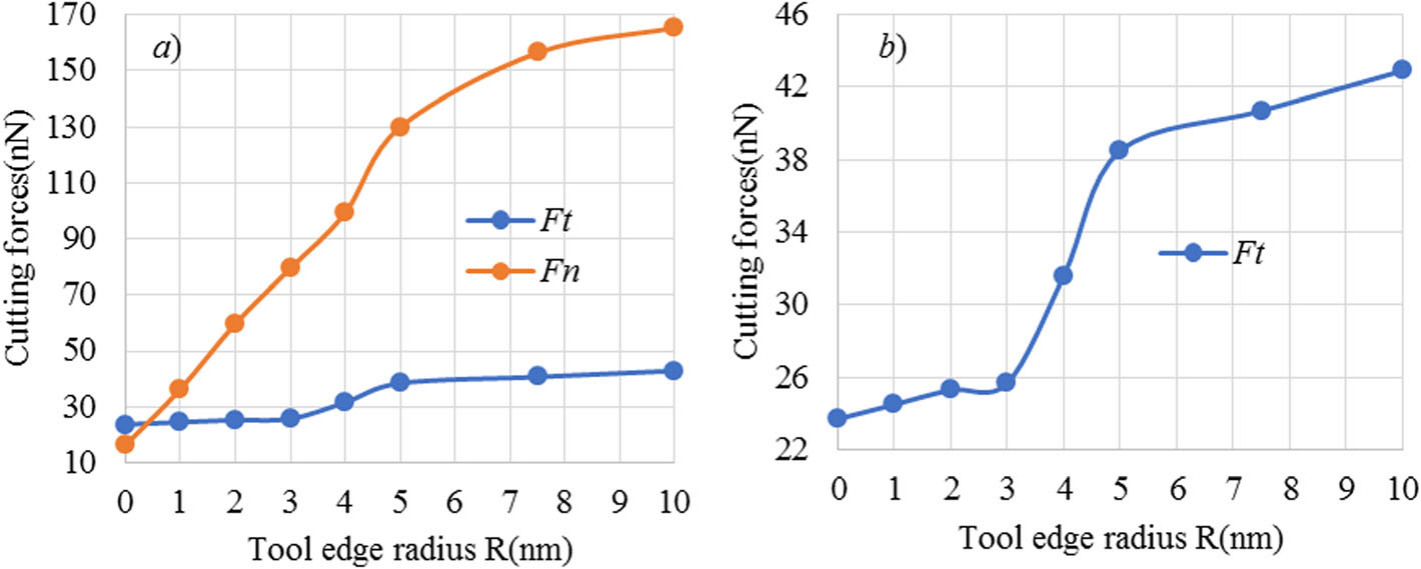
Plot of cutting forces versus cutting edge radius at cutting depth of 2 Å

Figure 12 shows the cutting-edge radius effect on the averaged cutting forces at cutting depth of 2 Å. Here, the tangential and normal components of cutting forces, namely, *F*_*t*_ and *F*_*n*_, are compared. As illustrated, for a sharp cutting tool, the ratio of *a/R* is 2.85, and the *F*_*t*_ of 16.4 nN is evidently smaller than *F*_*n*_ of 23.7 nN. As tool edge radius increases, both of *F*_*t*_ and *F*_*n*_ are increased. However, the *F*_*n*_ has exhibited greatly larger growth amplitudes than *F*_*t*_. It clearly indicates that as tool edge radius increases, the normal cutting force would have larger effect on material removal process at ACS cutting process. However, when tool edge radius is larger than about 3 nm, single atomic layer removal could not be achieved. Thus, a larger normal cutting force would not be helpful to enable cutting-based single atomic layer removal, and a lower normal cutting force should be preferably adopted.

As the normal cutting forces provide the compressive stress to enable elastic and/or plastic deformation on the processed surface, the tangential cutting forces would provide the shearing stress to chip formation. Therefore, it can be inferred that chip formation in cutting-based single atomic layer removal should be mainly driven by the tangential cutting force. It can be also seen in Fig. 12b that the *F*_*t*_ has exhibited three kinds of changes, which further leads to different surface topographies (see Fig. 6), including the following:

When tool edge radius is smaller than about 3 nm, namely, the ratio of *a/R* is 0.067, the *F*_*t*_ approximately remains unchanged, and it is always smaller than 25 nN, as edge radius increases. In such case, only one atomic layer is removed from workpiece surface.

When the tool edge radius is 3 nm and 5 nm, the ratios of *a/R* are 0.04 and 0.067, while *F*_*t*_ is evidently increased to about 38.5 nN. Consequently, more than one atomic layer is removed from workpiece surface, but there is small number of surface defects formed.

As for the edge radius of larger than 5 nm, *F*_*t*_ also gradually converges to a constant value. In this case, more than one atomic layer is removed. However, large numbers of surface defects are formed on the workpiece surface and subsurface.

It can be concluded that a very low tangential cutting force could enable cutting-based single atomic layer removal at a reasonable cutting-edge radius, such as 2 nm. In turn, single atomic layer removal could not be realized, despite of the larger tangential cutting force.

#### Influence of Atomic Sizing Effect

**Fig. 13:**
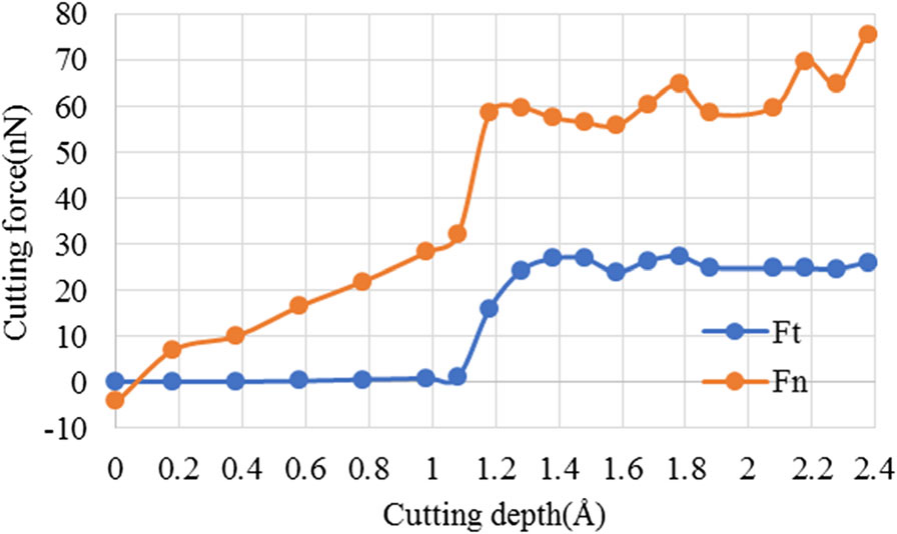
Plot of cutting forces versus cutting depth at edge radius of 2 nm

Figure 13 further gives the plot of cutting forces versus cutting depth at cutting edge radius of 2 nm. It can be found that at the cutting-edge radius of 2 nm, as cutting depth increases, both normal cutting force and tangential cutting forces have exhibited three-stage changes. There are evident changes at cutting depth of about 1.1 Å and 1.6 Å. The corresponding ratio of cutting depth (*a*) to workpiece atomic radius (*r*_*w*_) are 0.055 and 0.080, which are abovementioned two critical values of *a/r*_*w*_, C_1_ and C_2,_ as depicted in Section 3.1.

When cutting depth is smaller than 1.1 Å, namely, the ratio of *a/r*_*w*_ is less than 0.055, *F*_*t*_ is always 0 nN, while *F*_*n*_ is linearly increased to about 32 nN. However, the cutting forces could not enable plastic material deformation and removal on the workpiece surface. Consequently, there is only elastic deformation on workpiece surface, as shown in Fig. 3.

When the cutting depth is larger than 1.1 Å, but smaller than 1.6 Å, the ratio of *a/r*_*w*_ is more than 0.055 but less than 0.080. If this case, *F*_*t*_ has greatly increased from 0 nN to about 25 Nn, while *F*_*n*_ further increased to about 58 nN. The cutting forces are larger enough to enable material removal on workpiece surface. At the cutting depth of 1.4 Å, there is chip formation and material removal on workpiece surface. But the stable and continuous material removal could not be realized and many defects are formed on the processed surface, as shown in Fig. 4.

When the cutting depth is larger than 1.6 Å, both *F*_*t*_ and *F*_*n*_ are large enough to enable continuous material removal on workpiece surface. Consequently, chip is stably formed over the workpiece surface, and the targeted atomic layer is continuously removed via chip formation, forming a new processed surface, as shown in Fig. 5b and Fig. 6b.

Overall, both cutting-edge radius effect and atomic sizing effect have an influence on cutting force in cutting-based single atomic layer removal process, thereby changing the material removal and surface generation process.

## Discussions About Cutting-Based Single Atomic Layer Removal Mechanism

As per above analysis, both atomic sizing effect and cutting-edge radius effect have a great influence on the cutting-based single atomic layer removal mechanism. As summarized in Table 3, depending on the ratio of cutting depth to workpiece atomic radius(*a/r*_*w*_) and the ratio of cutting depth to edge radius(*a/R*), there are minimum 5 typical cases of material deformation and removal behaviours in cutting-based single atomic layer removal process. In this section, the fundamental material deformation and removal mechanism of each case is summarized.

**Table 3:** Effect of atomic sizing effect and cutting-edge effect on material removal during ACS cutting

		Ratio of cutting depth to atomic radius (*a/r*_*w*_)			
		(0, C1)	(C1, C2)	(C2, ∞)	
Ratio of cutting depth to edge radius (*a/R*)	(T1, ∞)	Elastic deformation, no material removal (case 1)	Noncontinuous material removal, chip formation (case 2)	Elastic deformation, chip formation by dislocation motion	Slip of 1 atomic layer (case 3)
	(T2, T1)				Slip of over 2 atomic layers (case 4)
	(0, T2)			No or extremely small chip formation, elastic-plastic deformation (case 5)	
Material removal behaviour		Sliding		Cutting	

In conventional machining, as cutting depth is significantly larger than the cutting-edge radius, the cutting-edge radius effect can be ignored. As the cutting depth is decreased to nanoscale, which is comparable or lower than the edge radius, the edge radius effect can be no longer ignored. In nanocutting, the material removal process is dominated by the extrusion deformation, which is greatly influenced by cutting-edge radius. As the cutting depth is further decreased to atomic scale, except for cutting-edge radius effect, a new sizing effect, atomic sizing effect [20] has a great influence on material removal.

**Fig. 14:**
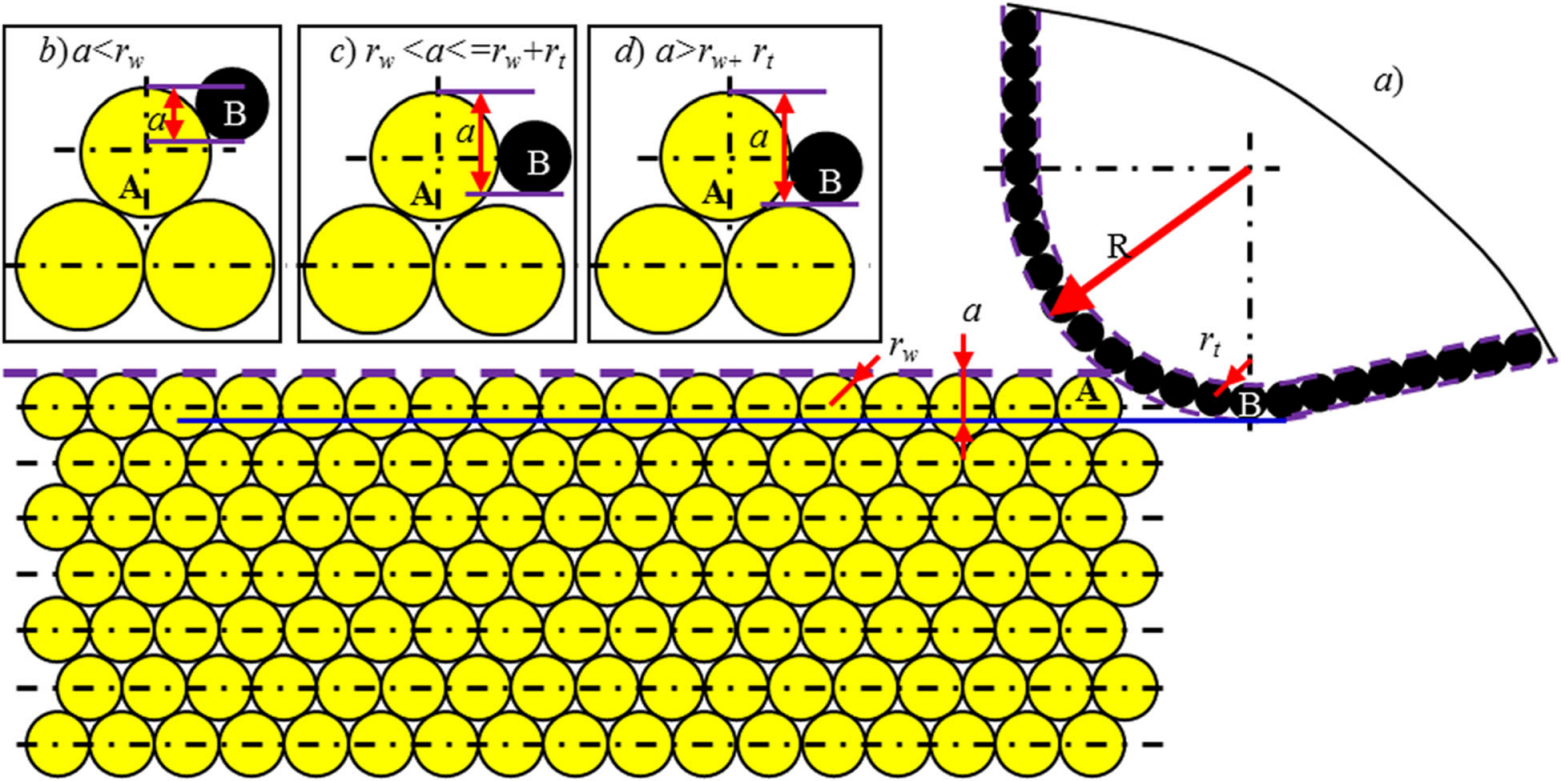
Schematic diagram for cutting-based single atomic layer removal

As shown in Fig. 14, in the cutting-based single atomic layer removal process, there are two portions involving in cutting, i.e., nanometric cutting edge and the lowest cutting tool atoms (*B*). Such two portions could be coupled to enable single atomic layer removal. The size of cutting edge, regarded as ‘nano-tool,’ is described by edge radius (*R*). The cutting edge is the envelope curve of the outermost atoms in cutting tool. Regarding the lowest atoms, as ‘atomic-tool,’ it is described by workpiece atomic radius (*r*_*w*_). The cutting-based single atomic layer removal is the results of the coupled actions of nano-tool and atomic-tool on workpiece material.

Firstly, depending on the ratio of cutting depth (*a*) to the workpiece atomic radius (*r*_*w*_), i.e., *a/r*, there are three kinds of material deformation behaviour in ACS cutting process.

1. The ratio of *a/r*_*w*_ is smaller than critical value 1(C_1_).

**Fig. 15:**
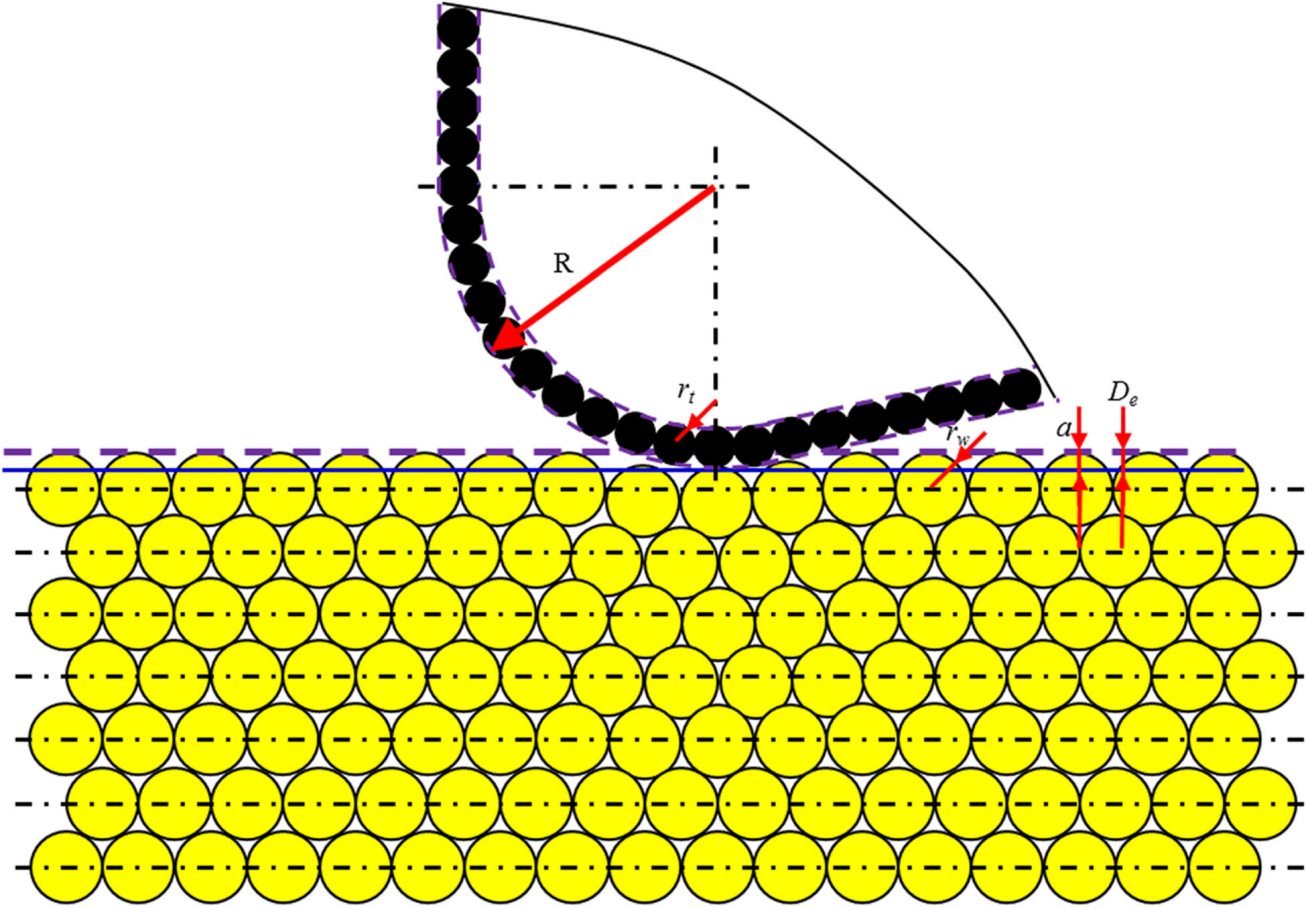
Schematic illustration of cutting-based single atomic layer removal at the ratio of a/r_w_ smaller than C1

As shown in Fig. 15, chip formation does not occur, but elastic deformation does occur on the workpiece surface. During cutting process, after workpiece passes the lowest point of cutting tool, the elastically deformed part would recover completely. Consequently, no material deformation and removal occurred on the topmost surface. In such case, the tool edge effect could be ignored. This ratio could be affected by material properties (case 1).

2. The ratio of *a/r*_*w*_ is larger than *C*_*1*_, but smaller than critical value 2 (C_2_).

**Fig. 16:**
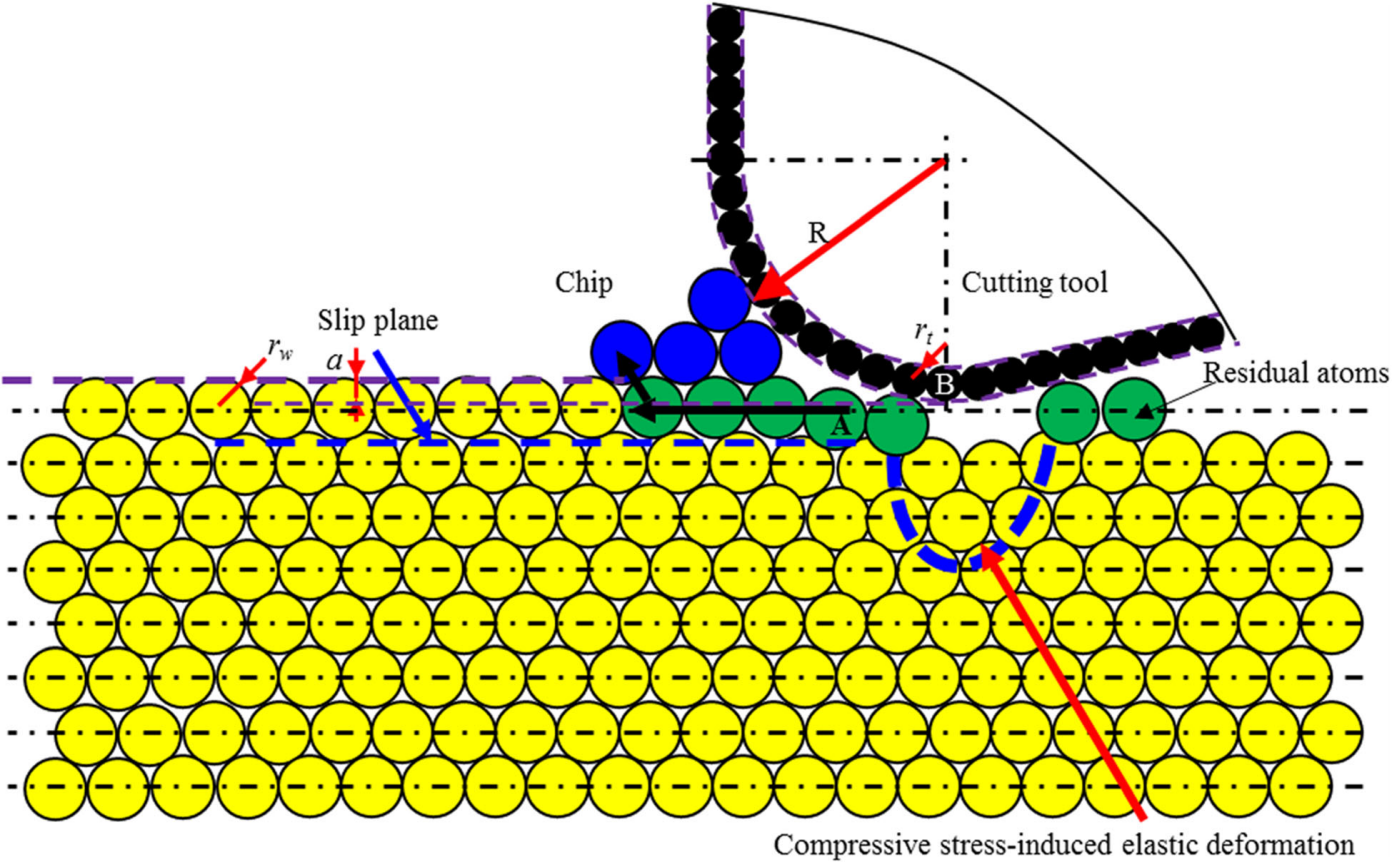
Schematic illustration of cutting-based single atomic layer removal at a/r_w_ larger than C_1_ and smaller than C_2_

As the ratio of the cutting depth to atomic radius (*a/r*_*w*_) increases to be larger than C_1_ but smaller than critical value 2(C_2_), there is material removal on the workpiece surface, but it is noncontinuous. In such case, only part of material within targeted atomic layer is formed into chip by shear stress-driven dislocation motion, while others are remained on the workpiece surface. When cutting tool passes over the workpiece surface, the surface quality of the processed surface is seriously deteriorated (case 2) (Fig. 16).

3. The ratio of *a/r*_*w*_ is larger than critical value 2 (C_2_).

When the ratio of cutting depth to atomic radius (*a/r*_*w*_) increases to be larger than critical value, there is a continuous material removal by chip formation during cutting process. In such case, the edge radius effects can no longer be ignored. For ACS cutting process, the maximum cutting depth is subnanometer order. At such extremely low cutting depth, regardless of the nominal rake angle, the effective rake angle is always largely negative. The negative rake face can produce the necessary shear stress to enable chip formation by dislocation motion and the compressive force to enable the elastic and/or plastic deformation on the processed surface.

Depending on the ratio of cutting depth (*a*) to tool edge radius (*R*), different kinds of elastic and/or plastic deformation process occur on the workpiece surface. There are two threshold values of *a/R*, namely, threshold value 1 (*T*_*1*_) and threshold value 2 (*T*_2_), leading to different material removal processes.

- The ratio of *a/R* is larger than threshold (*T*_*1*_).

**Fig. 17:**
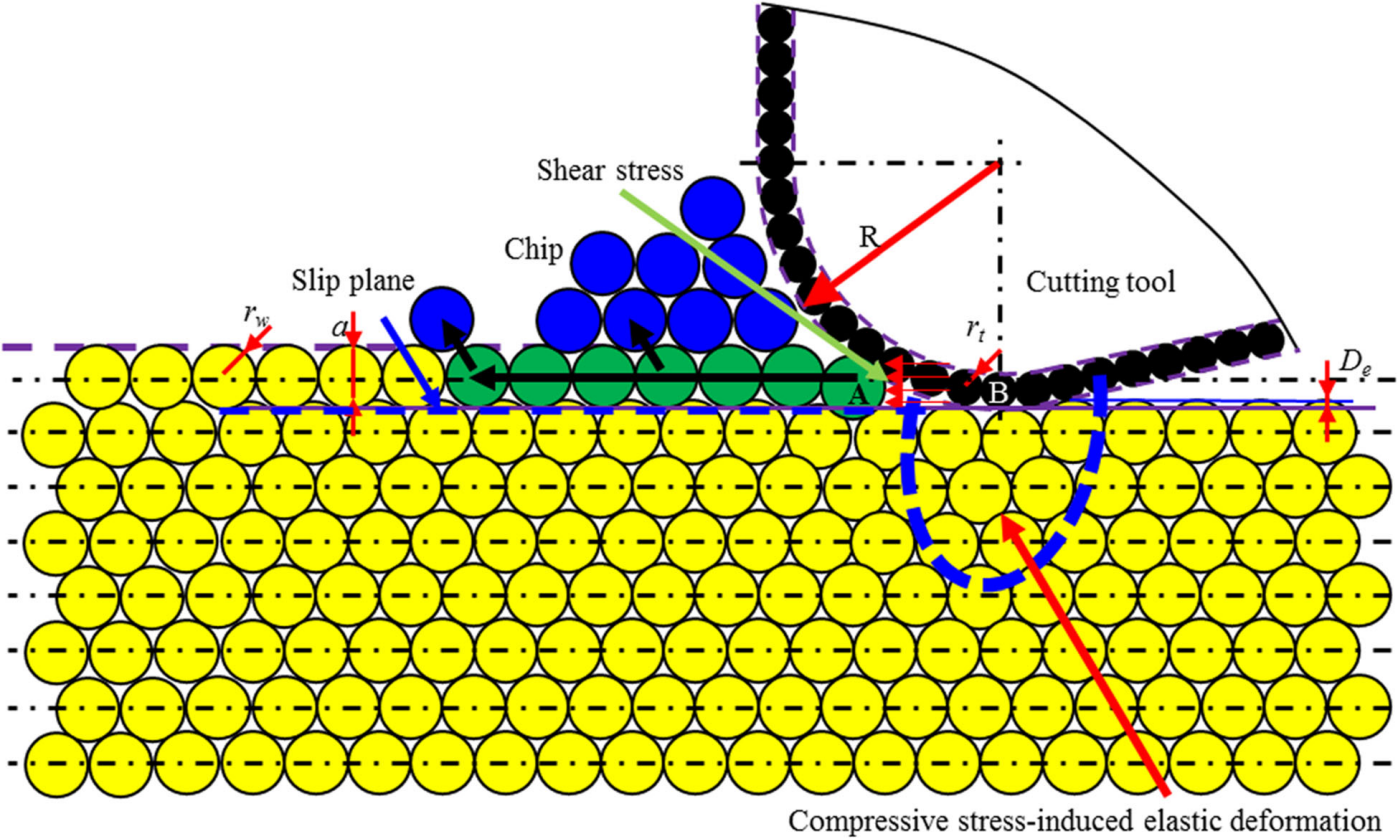
Schematic illustration of cutting-based single atomic layer removal at a/r_w_ larger than C_2_ and a/R larger than T_1_

As shown in Fig. 17, there is one atomic slip plane. The workpiece material below this plane would have an elastic deformation. As for the materials above this slip plane, it undergoes a plastic deformation by dislocation motion. A part of materials is also formed into chip by shearing stress-driven dislocation motion, while the other undergoes dislocation slip. After the workpiece passes the lowest point of the cutting tool, the elastically deformed part recovers completely (case 3).

b. The ratio of *a/R* is lower than threshold 1 (*T*_1_), but larger than threshold 2 (*T*_2_).

**Fig. 18:**
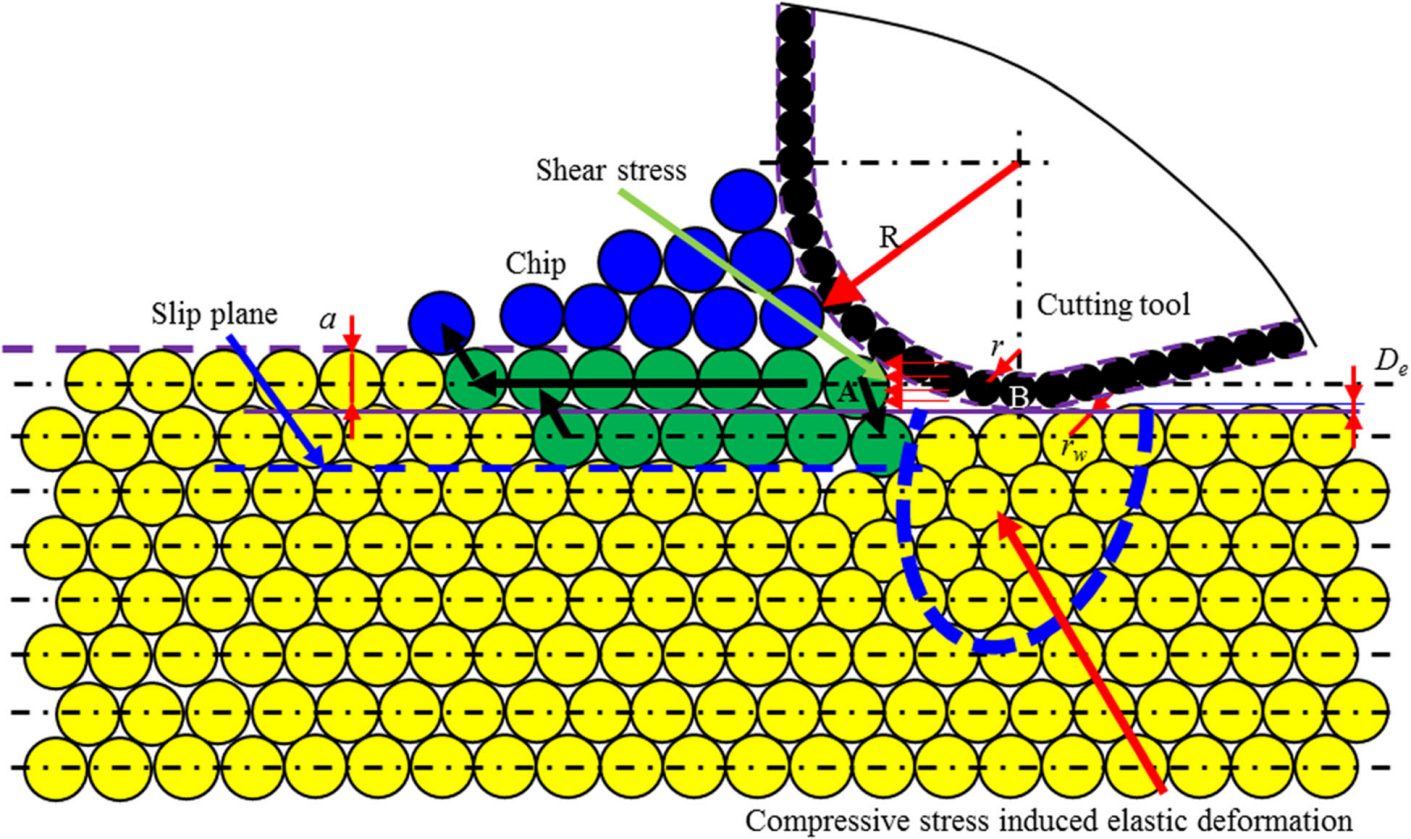
Schematic illustration of cutting-based single atomic layer removal at a/r_w_ larger than C_2_, a/R larger than T_2_, smaller than T_1_

Under the action of cutting edge, minimum two atomic layers undergo dislocation slip, while elastic deformation occurs on the processed surface, as shown in Fig. 18. The cutting edge provides a force to generate the shear stress to enable chip formation by dislocation motion and compressive stress to induce elastic deformation on the processed surface. In such case, part of material within the targeted atomic layer is formed into chip by dislocation motion, while the other is extruded into other atomic layers to form new processed surface. It also drives the slip of other atomic layers on the workpiece surface, leading to the negative dislocation climb. After workpiece material passes the lowest point of the cutting tool, the elastic portion springs back (case 4).

c. The ratio of *a/R* is lower than threshold 2 (*T*_2_).

**Fig. 19:**
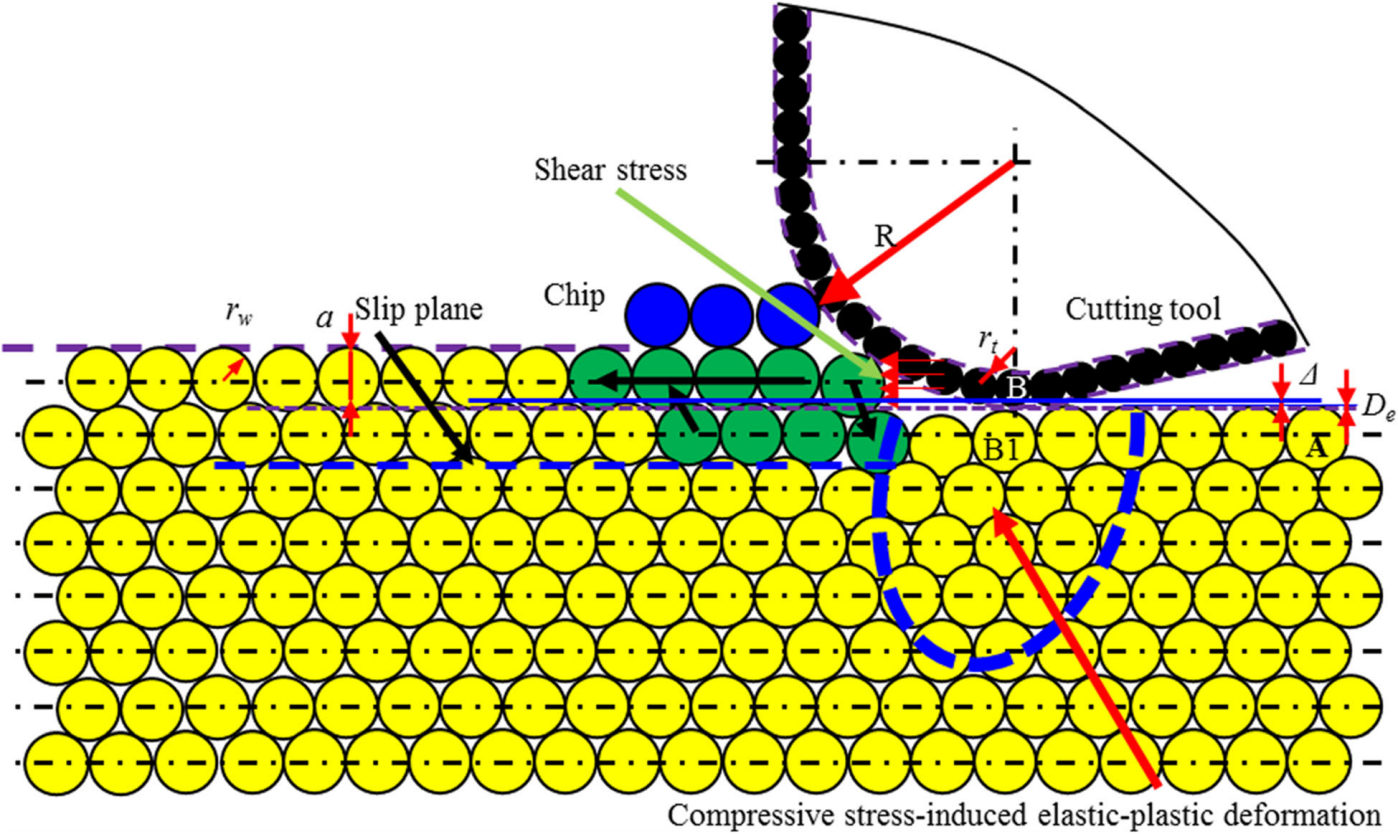
Schematic illustration of cutting-based single atomic layer removal at a/r larger than C_2_ and a/R lower than T_2_

As the ratio of *a/R* decreases to lower than *T*_2_, there is either no chip formation or extremely small volume of chip formation, but elastic-plastic deformation occurs on the processed surface, as shown in Fig. 19. After workpiece material passes the lowest point of cutting edge, the elastic deformed part springs back. The plastic deformed part (*Δ*) leads to lasting deformation. Such ratio is related with material properties, tool geometry and process conditions (case 5).

## Conclusions

In the present study, both of atom sizing effect and cutting-edge radius effect are analysed to investigate their influence on chip formation, surface generation, subsurface deformation, and atomic displacement behaviour during the cutting of monocrystalline copper towards single atomic layer removal. The following conclusions can be drawn.

1. A new model is proposed to describe the underlying material deformation and removal mechanism in the cutting-based single atomic layer removal process, which exhibits four characteristics, including chip formation by dislocation motion, elastic deformation occurring on the processed surface, atomic sizing effect, and cutting-edge radius effect.
2. Both of atomic sizing effect and cutting-edge radius effect have a great influence on the material deformation and removal during the cutting process of monocrystalline copper towards single atomic layer removal. With a specific ratio of cutting depth to workpiece atom radius (*a/r*_*w*_) and that of cutting depth to edge radius (*a/R*), cutting-based single atomic layer removal could be achieved on Cu (111) surface.
3. Chip formation is affected by the ratios of *a/r*_*w*_ and *a/R*. There is a chip formation only when the ratio of *a/r*_*w*_ is larger than one critical value (C1) and the ratio of *a/R* is smaller than one threshold value (T1). Moreover, chip formation is mainly dependent on the shear stress-driven dislocation motion, significantly different from the extrusion-dominated chip formation in nanocutting and shearing-dominated chip formation in conventional machining.
4. Single atomic layer removal can be achieved via layer-by-layer removal and multi-layer removal. The former one refers to that the targeted atomic layer could be either fully removed from workpiece surface. The latter one means that the first atomic layer is partly removed while the remaining materials are pressed into other atomic layers, forming a new processed surface.
5. There is only elastic deformation occurring on the processed surface during ACS cutting process, different from the elastic-plastic deformation in nanocutting. It can be regarded as one characteristic feature in ACS cutting.
6. Depending on the combined effect of atom sizing effect and cutting-edge radius effect, there exist five cases of material deformation and removal processes during the cutting-based single atomic layer removal, i.e., no workpiece material is removed (case 1), workpiece materials are non-continuously removed (case 2), a part of materials is formed into chip while others undergoes material slip via dislocation motion (case 3), a part of materials within the targeted atomic layer is formed into chip while others are extruded into other atomic layers to form new processed surface (case 4), and the elastic deformed part springs back, while the plastic deformed part leads to a lasting deformation (case 5).

## Data Availability

Authors declare that the materials, data, and associated protocols are available to the readers, and all the data used for the analysis are included in this article.

## Abbreviations

ACS: Atomic and close-to-atomic scale
ACSM: Atomic and close-to-atomic scale manufacturing
MD: Molecular dynamics
